# The Pattern of Fractures in Road Traffic Crashes: Findings From the National Trauma Registry in Iran

**DOI:** 10.34172/jrhs.8915

**Published:** 2025-06-10

**Authors:** Mahgol Sadat Hassan Zadeh Tabatabaei, Mohammad Soleimani, Seyyed Hossein Shafiei, Mohammadreza Zafarghandi, Vafa Rahimi-Movaghar, Vali Baigi, Esmaeil Fakharian, Seyed Houssein Saeed-Banadaky, Vahid Hoseinpour, Homayoun Sadeghi-Bazargani, Reza Farahmand Rad, Farideh Sadeghian, Mehdi Nasr Isfahani, Vahid Rahmanian, Amir Ghadiphasha, Mohammad Shahidi, Mohamad Kogani, Sobhan Pourmasjedi, Seyed Mohammad Piri, Sara Mirzamohamadi, Armin Khavandegar, Khatereh Naghdi, Payman Salamati

**Affiliations:** ^1^Sina Trauma and Surgery Research Center, Sina Hospital, Tehran University of Medical Sciences, Tehran, Iran; ^2^Orthopedic Subspecialty Research Centre, Tehran University of Medical Science, Tehran, Iran; ^3^Trauma Research Center, Kashan University of Medical Sciences, Kashan, Iran; ^4^Trauma Research Center, Rahnemoon Hospital, School of Medicine, Shahid Sadoughi University of Medical Sciences, Yazd, Iran; ^5^Department of Emergency Medicine, School of Medicine, Urmia University of Medical Sciences, Urmia, Iran; ^6^Road Traffic Injury Research Center, Tabriz University of Medical Sciences, Tabriz, Iran; ^7^Clinical Research Development Center, Imam Ali and Taleghani Hospital, Kermanshah University of Medical Sciences, Kermanshah, Iran; ^8^Center for Health-Related Social and Behavioral Sciences Research, Shahroud University of Medical Sciences, Shoahroud, Iran; ^9^Department of Emergency Medicine, Faculty of Medicine, Isfahan University of Medical Sciences, Isfahan, Iran; ^10^Trauma Data Registration Center, Isfahan University of Medical Sciences, Isfahan, Iran; ^11^Research Center for Social Determinants of Health, Jahrom University of Medical Sciences, Jahrom, Iran; ^12^Shahid Modarres Hospital, Saveh University of Medical Sciences, Tehran, Iran; ^13^Department of Emergency Medicine, Qom University of Medical Sciences, Qom, Iran; ^14^Research Center for Environmental Contaminants, Abadan University of Medical Sciences, Abadan, Iran; ^15^Research Center for War-Affected People, Tehran University of Medical Sciences, Tehran, Iran

**Keywords:** Accidents, Traffic, Wounds and injuries, Fractures, Bone, Pedestrians

## Abstract

**Background:** Fractures constitute a significant concern in low-income and middle-income countries, primarily due to road traffic crashes (RTCs), a leading cause of such injuries. This study aimed to analyze fracture patterns resulting from RTCs in Iran.

**Study Design:** A cross-sectional study.

**Methods:** A registry-based study was conducted using data from the National Trauma Registry of Iran spanning 2016–2023. The study included 10,114 trauma patients involved in RTCs, encompassing car and motorcycle crashes with at least one fracture. International Classification of Diseases (ICD-10) codes were used for data analysis, considering both orthopedic and non-orthopedic admissions related to RTCs. Fracture incidence was compared among pedestrians, drivers/riders, and passengers/pillions.

**Results:** Males constituted a significant majority of the car (90.1% drivers, 72.1% pedestrians, and 47.0% passengers) and motorcycle (99.6% riders, 77.0% pedestrians, and 65.3% pillions) crashes (*P*<0.001). Patients under 18 comprised 18.4% of the motorcycle riders and 2.5% of the car drivers. Drivers showed the highest frequency of head injuries (26.9%, *P*<0.010), while pedestrians had the highest frequency of upper extremity injuries (73.1%, *P*<0.001). Drivers also demonstrated a higher frequency of vertebral fractures than passengers (C3-C7: 3.2% vs. 1.4%, *P*=0.006). Riders (33.5%) displayed a higher frequency of head and face fractures compared to pillions (24.8%) and pedestrians (17.4%) (Head: pedestrian vs. rider, *P*<0.001; pedestrian vs. pillion, *P*=0.018; rider vs. pillion, *P*=0.005; Face: pedestrian vs. rider, *P*<0.001; pedestrian vs. pillion, *P*<0.001; rider vs. pillion, *P*=0.033).

**Conclusion:** The study provided valuable information on the fracture patterns associated with RTCs among road user groups.

## Background

 Road traffic crashes (RTCs), ranking as the ninth leading cause of death globally (and the second cause of death in Iran), constitute a significant public health concern.^1,2^ The global road safety report of the World Health Organization indicated that Iran recorded a substantial rate of RTC-related fatalities (N = 22 918) between 2007 and 2008.^3^ Beyond the loss of life, these incidents cause an estimated 20–50 million non-fatal injuries each year, many of which result in long-term disabilities. RTCs accounted for 1.06% of all years lived with disability and 5.63% of disability-adjusted life years among the Iranian population in 2019, according to the Global Burden of Diseases Report.^4,5^

 In low-income countries, RTCs are a leading cause of fractures, with survivors and their families facing significant social, physical, and psychological challenges.^6^ Families often struggle with ongoing medical costs and the financial support needed for disabled individuals. Furthermore, injuries can lead to temporary unemployment or early retirement, creating a burden on both insurance companies and society.^7^

 While numerous epidemiological studies conducted in Iran have focused on various aspects of RTCs (e.g., injury patterns, mortality rates, demographic characteristics of patients, and associated costs), less attention has been given to the patterns of fractures in RTC patients.^8-12^ Further, insufficient information exists regarding variations in fracture patterns based on the occupant’s seating position and road participation.^13^ Understanding the patterns of RTC-related fractures is paramount for policymakers and strategic planners. This can significantly benefit public emergency services, healthcare facilities, and rehabilitation centers by helping them prioritize and implement preventive measures and improve their quality of care.^14^ Moreover, detailed data on fracture patterns occurring in different car seat locations can draw attention to specific safety requirements and propose enhancements for safety features in those particular areas of the vehicle.^13^

 This study aims to determine the pattern of RTC-associated fractures in drivers, riders, passengers, pillions, and pedestrians involved in car and motorcycle crashes for eight years in Iran.

## Methods

###  Study design and participants

 This registry-based study was conducted among trauma patients associated with the National Trauma Registry of Iran (NTRI) between September 17, 2016, and February 1, 2023. This registry, a collaborative initiative involving multiple major trauma centers across the country, was initially established at Sina Hospital in Tehran, Iran, in 2015 by the Sina Trauma and Surgery Research Center. Previous articles have provided detailed information about the NTRI’s registration process, a minimal dataset, and data quality assurance.^15^ The study collects comprehensive information on trauma patients from the 12 major trauma centers currently active across the country, including Sina hospital in Tehran (n = 1611), Al-Zahra hospital in Isfahan (n = 468), Imam Hossein hospital in Shahroud (n = 885), Imam Khomeini hospital in Urmia (n = 1249), and Peymanie hospital in Jahrom (n = 215). The other trauma centers are Shohada hospital in Tabriz (n = 1172), Shahid Beheshti hospital in Qom (n = 110), Shahid Beheshti hospital in Kashan (n = 2376), Shahid Rahnemoun hospital in Yazd (n = 838), Shahid Modarres hospital in Saveh (n = 507), Shahid Beheshti hospital in Abadan (n = 27), and Taleghani hospital in Kermanshah (n = 656). The study encompassed all trauma patients involved in RTCs, including car and motorcycle crashes, who presented with at least one fracture site and met the NTRI’s inclusion criteria. These criteria included hospitalization for more than 24 hours, fatal injuries on the first day of admission, or transfer from other hospitals’ intensive care units (ICUs). All RTCs involving bicycles, pick-up trucks or vans, buses, and heavy transport vehicles were excluded from the study.

###  The National Trauma Registry of Iran data collection

 The data collection process in the NTRI involves several key stages. First, trauma patients are identified upon admission to participating trauma centers. They include patients hospitalized for more than 24 hours, those who have died in the emergency department after being hospitalized for less than 24 hours, and those transferred from the ICU of a previous hospital to the ICU of the current hospital, also with less than 24 hours. Next, trained medical staff collect relevant patient information using a 99-item questionnaire. This questionnaire includes demographics (18 variables), injury-related information (20 variables), pre-hospital information (22 variables), emergency department information (23 variables), hospital procedures (2 variables), diagnosis (2 variables), outcomes (6 variables), financial aspects (2 variables), and injury severity (3 variables). Standardized forms are used to ensure consistency across different centers. The data are recorded by the registrars through interviews, physical exams, medical records, and hospital information systems. The collected data are then inputted into a centralized database web portal (www.ntriran.ir). This portal was created using the programming language “C#.net 4” and the server software “SQL-server 2012 r2”. Regular audits and quality checks are performed by trained supervisors, who are professional physicians, to ensure the accuracy, consistency, and completeness of the data entered into the registry. Additionally, a separate evaluator, who is a surgeon, examines the precision of injury severity data according to established standards. These standards, released by the Association for the Advancement of Automotive Medicine, involve assessing the abbreviated injury scale, the abbreviated injury scale pre-dot code, and the injury severity score (ISS). Finally, the data become available for analysis after compilation, allowing researchers to examine trends, injury patterns, and outcomes among trauma patients. Previous articles extensively addressed the data collection process during the NTRI pilot phase.^15-23^

###  Variables

 The data analysis was conducted using the International Classification of Diseases and Causes of Death (ICD-10) codes for orthopedic and non-orthopedic admissions associated with RTCs. For orthopedic admissions, the primary focus was on the fractures of the extremities, neck, thoracic spine, lumbar spine, and pelvis. On the other hand, the fractures of the skull, facial bones, ribs, and sternum were examined for non-orthopedic admissions. Fractures were classified according to the location of the injury, encompassing skull and facial bone fractures (S02), neck fractures (S12), rib, sternum, and thoracic spine fractures (S22), and lumbar spine and pelvis fractures (S32). The other types included shoulder and upper arm fractures (S42), forearm fractures (S52), wrist and hand fractures (S62), femur fractures (S72), lower leg (including ankle) fractures (S82), and foot (except ankle) fractures (S92). The fractures were also grouped and featured based on the topography of the fractures in upper and lower limbs as shoulder (S42.0, S42.1, and S42.2), arm (S42.3), elbow (S42.4, S52.0, and S52.1), forearm (S52.2, S52.3, and S52.4), wrist (S52.5, S52.6, and S62.0), and hand (S62.1, S62.2, S62.3, S62.4, S62.5, S62.6, and S62.7). The remaining fractures were related to the pelvis (S32.1, S32.2, S32.3, S32.4, and S32.5), hip (S72.0 and S72.1), thigh (S72.2 and S72.3), knee (S72.4, S82.0, and S82.1), leg (S82.2 and S82.4), ankle (S82.3, S82.5, and S82.6), and foot (S92, S92.0, S92.1, S92.2, S92.3, S92.4, S92.5, S92.7, and S92.9). Variables analyzed in this study included gender, age group, transport to hospital, type of accident, number of fractures, body region of fractures, and fracture sites. The patients were transported to the hospital by an ambulance, private vehicles, and others (i.e., police, public transportation, and on foot). The number of registered fractures pertained to the total number of fracture lines, regardless of whether a single line affected two or more bones. When a single bone exhibited two distinct fracture lines, each line was individually tallied based on the number of fracture lines observed. Comminuted fractures were grouped with multiple fractures.

###  Statistical analysis

 Numbers and percentages were used for describing nominal and categorical variables. The proportion of nominal and categorical variables among pedestrians, drivers/riders, and passengers/pillions was compared using the Chi-square test. In addition, this test was utilized to compare the types of fractures among pedestrians, drivers/riders, and passengers/pillions. Further, the Bonferroni approach was applied to correct multiple comparisons.

 Logistic regression models and “margins” command were employed to estimate the adjusted body region of fracture, clinical management and outcomes, and fracture frequency by three groups. The “margins” command in STATA was employed for age, gender, safety device, ISS, type of vehicle (car or motorcycle), and type of accident adjustment. First, some logistic regression models were fitted. The body region of fracture, clinical management and outcomes, and types of fractures were considered dependent variables in logistic regression models. Then, adjusted probabilities were estimated using the mentioned command.

 A *P* value less than 0.05 was considered statistically significant. STATA software (version 14.0, STATA Corporation, College Station, TX) was used for data analysis.

## Results

 Overall, 10 114 cases of trauma patients associated with RTCs were evaluated, comprising 4755 (47.0%) car crashes and 5359 (53.0%) motorcycle crashes. In general, 921, 2768, 2039, 2720, and 1633 crashes were recorded in 2018, 2019, 2020, 2021, and 2022, respectively. The average age of individuals involved in car crashes was 37.3 ± 19.3 years, while for motorcycle crashes, it was 33.7 ± 16.8 years (*P*< 0.001).

 In car crashes, 796 (47.0%) car passengers were male. Male individuals constituted 1243 (72.1%) pedestrians struck by cars and 1205 (90.1%) drivers (*P* < 0.001). Notably, 483 (28.0%) pedestrians and 240 (18.0%) drivers in car crashes were transported to the hospital by private cars (*P* < 0.001). Approximately 583 (43.6%) drivers had multiple or comminuted fractures, significantly more than 413 (24.0%) pedestrians injured in car crashes and 651 (38.4%) car passengers (*P*< 0.001).

 Male individuals constituted 396 (77.0%) pedestrians struck by motorcycles, 4,193 (99.6%) riders, and 416 (65.3%) motorcycle pillions (*P*< 0.001). Almost 773 (18.4%) riders and 230 (36.2%) pillions were children under the age of 18 in motorcycle crashes (*P*< 0.001). Pedestrians over the age of 64 demonstrated a significantly higher crash rate (17.1%) compared to riders (4.6%) and pillions (3.3%) in the same age group in motorcycle crashes (*P*< 0.001). Collision crashes involved 3350 (80.6%) riders and 433 (70.0%) motorcycle pillions, while rollovers accounted for 807 (19.4%) riders and 186 (30.0%) motorcycle pillions (*P*< 0.001). In motorcycle crashes, more riders (28.9%) experienced multiple and comminuted fractures compared to pedestrians struck by motorcycles (18.5%) (*P*< 0.001). Additional demographic characteristics and crash-related details of RTC patients are provided in Table 1.

**Table 1:** Demographic and crash-related characteristics of road traffic crash patients by road participation

**Variables**	**Car crashes**				**Motorcycle Crashes**			
	**Pedestrian (n=1724)**	**Driver (n=1337)**	**Passenger (n=1694)**	***P *** **value**	**Pedestrian (n=514)**	**Rider (n=4208)**	**Pillion (n=637)**	***P *** **value**
Gender				0.001				0.001
Male	1243	1205	796		396	4193	416	
Female	481	132	898		118	15	221	
Age group				0.001				0.001
≤ 18	382	33	368		103	773	230	
19-64	1057	1247	1203		323	3239	384	
≥ 65	285	57	123		88	192	22	
Transport to hospital				0.001				0.979
Ambulance	1232	1078	1370		431	3530	529	
Private vehicle	483	240	293		80	662	105	
Others	8	19	30		2	12	2	
Type of accident				0.432				0.001
Collision	NA	788	972		NA	3350	433	
Rollover	NA	546	714		NA	807	186	
Number of fractures				0.001				0.001
Isolated fractures	1311	754	1043		419	2991	475	
Multiple/comminuted fractures	413	583	651		95	1217	162	

*Note*. NA: Not applicable

 In car crashes, both drivers and passengers exhibited a heightened frequency of fractures in various body regions, including the face, pelvis, spine, and lower extremities, in comparison to pedestrians (*P*< 0.001). Notably, drivers had the highest frequency of head injuries (26.9%), followed by car passengers (21.2%) and pedestrians struck by cars (12.1%) (*P*< 0.010). Neck injuries were less common, with 1.8% of drivers and 1.6% of car passengers experiencing them, while only 0.6% of pedestrians struck by cars suffered from neck injuries (*P*= 0.010). Thorax injuries were most frequent in drivers (17.1%), followed by car passengers (12.6%) and pedestrians involved in car crashes (3.6%) (*P*< 0.010). Upper extremity injuries were significantly more prevalent in pedestrians struck by cars (73.1%) compared to drivers (43.5%) and car passengers (43.0%) (*P*< 0.001).

 Shifting to motorcycle crashes, riders (20.9%) displayed a significantly higher frequency of head fractures in comparison to pillions (15.5%) and pedestrians (10.9%) (pedestrian vs. rider, *P*< 0.001; pedestrian vs. pillion, *P*= 0.018; rider vs. pillion, *P*= 0.005). Additionally, 12.6% of riders, 9.3% of pillions, and 6.5% of pedestrians struck by motorcycles experienced face fractures (pedestrian vs. rider, *P*< 0.001; pedestrian vs. pillion, *P*< 0.001; rider vs. pillion, *P*= 0.033). Thorax injuries were more frequent in riders (4.8%) and motorcycle pillions (4.0%) compared to pedestrians struck by motorcycles (1.1%) (*P*< 0.010). Moreover, spine traumas were significantly higher in riders (5.6%) and motorcycle pillions (5.6%) than in pedestrians struck by motorcycles (1.2%) (*P*< 0.001). Upper extremity injuries were also more prevalent in pedestrians struck by motorcycles (67.1%) than in riders (61.9%) (*P*= 0.030). Table 2 details the distribution of fractures across various body regions among individuals involved in RTCs, adjusted for age, gender, and safety device.

**Table 2:** Body region in the fractures of road traffic crash patients by road participation adjusted for age, gender, and safety device^*^

**Variables**	**Car crashes**				**Motorcycle crashes**			
	**Pedestrian**	**Driver**	**Passenger**	**Pairwise** **comparison**	**Pedestrian**	**Rider**	**Pillion**	**Pairwise** **comparison**
Head	12.1 (10.6, 13.7)	26.9 (24.2, 29.6)	21.2 (19.1, 23.2)	A < C < B	10.9 (8.3, 13.5)	20.9 (19.6, 22.2)	15.5 (12.5, 18.6)	B > C > A
Face	6.9 (5.7, 8.1)	17.4 (15.2, 19.6)	15.8 (14.0, 17.7)	B > A < C	6.5 (4.3, 8.6)	12.6 (11.6, 13.7)	9.3 (6.9, 11.7)	B > C > A
Neck	0.6 (0.2, 1.0)	1.8 (0.9, 2.7)	1.6 (1.0, 2.1)	B > A < C	0.5 (-0.1, 1.1)	1.1 (0.7, 1.4)	1.0 (0.2, 1.7)	-
Thorax	3.6 (2.7, 4.5)	17.1 (14.8, 19.4)	12.6 (10.9, 14.3)	A < C < B	1.1 (0.3, 2.0)	4.8 (4.1, 5.5)	4.0 (2.2, 5.7)	B > A < C
Pelvis	1.8 (1.2, 2.4)	5.2 (3.8, 6.7)	4.3 (3.3, 5.3)	B > A < C	1.7 (0.5, 3.0)	1.6 (1.2, 2.0)	1.6 (0.6, 2.7)	-
Spine	4.5 (3.6, 5.5)	20.2 (17.7, 22.7)	14.6 (12.8, 16.4)	A < C < B	1.2 (4.8, 6.4)	5.6 (4.8, 6.4)	5.6 (3.6, 7.5)	B > A < C
Upper extremity	73.1 (70.8, 75.3)	43.5 (40.6, 46.4)	43.0 (40.5, 45.5)	C < A > B	67.1 (62.9, 71.3)	61.9 (60.4, 63.5)	64.4 (60.3, 68.5)	A > B
Lower extremity	29.9 (27.6, 32.2)	43.2 (40.3, 46.1)	46.5 (44.0, 49.0)	B > A < C	32.9 (28.7, 37.1)	35.4 (33.9, 36.9)	33.8 (29.7, 37.8)	-
Multiple traumas	22.9 (20.9, 24.9)	45.9 (42.9, 48.8)	38.2 (35.8, 40.6)	A < C < B	16.6 (13.5, 19.8)	29.4 (28.0, 30.9)	24.6 (21.0, 28.2)	B > C > A

*Note*. A: Pedestrian; B: Driver/rider; C: Passenger/pillion. ^*^The presented values are adjusted body region of fracture frequencies estimated using the “margins” command in STATA.

Figures S1 and S2 (Supplementary file 1) delineate how fractures are distributed among pedestrians struck by cars, drivers, and car passengers, with a specific focus on the upper and lower limbs. Meanwhile, Figures S3 and S4 (Supplementary file 1) present similar data for motorcycle accidents.

 In car crashes, the need for open reduction was observed in 47.5% of pedestrians struck by cars, with slightly lower percentages for car passengers (42.5%) and drivers (39.6%) (*P*< 0.010). The frequency of spinal repair demonstrated a similar trend, being highest among drivers (5.6%), followed by car passengers (3.6%) and pedestrians involved in car crashes (1.0%) (pedestrian vs. driver, *P*< 0.001; pedestrian vs. passenger, *P*< 0.001; driver vs. passenger, *P*= 0.033). In motorcycle crashes, a parallel pattern emerged concerning spinal repair. Riders displayed the highest rate of spinal repair (1.7%), surpassing pillions (0.4%) and pedestrians (0.2%) (pedestrian vs. rider, *P*= 0.010; rider vs. pillion, *P*= 0.034). Table 3 depicts a comprehensive overview of clinical management and outcomes in RTC-related trauma patients adjusted for age, gender, and safety device.

**Table 3:** Clinical management and outcomes of road traffic crash patients by road participation adjusted for age, gender, and safety device^*^

**Variables**	**Car crashes**				**Motorcycle crashes**			
	**Pedestrian**	**Driver**	**Passenger**	**Pairwise** **comparison**	**Pedestrian**	**Rider**	**Pillion**	**Pairwise** **comparison**
Craniotomy	0.7 (0.3, 1.1)	2.1 (1.2, 3.0)	1.1 (0.6, 1.7)	B > A	0.3 (-0.1, 0.8)	1.2 (0.9, 1.6)	2.2 (0.9, 3.6)	C > A
Plaster/splints	40.4 (37.9, 42.9)	33.8 (31.0, 36.6)	32.6 (30.3, 34.9)	C < A > B	50.2 (45.7, 54.7)	49.8 (48.3, 51.4)	45.6 (41.4, 49.9)	-
Traction	2.2 (1.5, 2.9)	4.4 (3.1, 5.6)	3.9 (2.9, 4.9)	B > A < C	3.0 (1.5, 4.6)	2.7 (2.2, 3.2)	1.6 (0.6, 2.7)	-
Fracture immobilization	39.8 (37.4, 42.2)	42.5 (39.6, 45.4)	38.1 (35.7, 40.6)	B > C	54.1 (49.7, 58.5)	46.0 (44.5, 47.6)	46.6 (42.3, 50.8)	C < A > B
Closed reduction	38.7 (36.2, 41.2)	30.4 (27.8, 33.1)	36.7 (34.3, 39.1)	A > B < C	40.2 (35.7, 44.6)	33.2 (31.7, 44.6)	31.5 (27.5, 35.4)	C < A > B
Open reduction	47.5 (45.0, 50.0)	39.6 (36.8, 42.5)	42.5 (40.0, 44.9)	C < A > B	47.3 (42.8, 51.8)	48.0 (46.4, 49.6)	50.3 (46.0, 54.6)	-
Amputation	0.1 (-0.1, 0.2)	-	0.2 (-0.2, 0.5)	-	-	0.3 (0.1, 0.4)	0.2 (-0.2, 0.7)	-
Spinal repair	1.0 (0.5, 1.4)	5.6 (4.1, 7.1)	3.6 (2.7, 4.6)	B > C > A	0.2 (-0.1, 0.6)	1.7 (1.0, 2.4)	0.4 (0.0, 0.9)	C < B > A
Mechanical ventilation	3.6 (2.7, 4.4)	7.9 (6.3, 9.4)	7.1 (5.8, 8.5)	B > A < C	1.8 (0.7, 2.9)	3.9 (3.3, 4.5)	3.3 (1.7, 4.9)	B > A
ICU admission	11.3 (9.8, 12.8)	20.3 (17.9, 22.7)	18.4 (16.4, 20.3)	B > A > C	7.4 (5.2, 9.5)	10.4 (9.4, 11.4)	10.0 (7.3, 12.7)	B > A
Death	1.2 (0.7, 1.7)	1.8 (1.0, 2.6)	2.0 (1.2, 2.8)	-	0.4 (-0.1, 0.8)	0.9 (0.6, 1.3)	0.9 (-.0.1, 1.9)	-

*Note*. A: Pedestrian; B: Driver/rider; C: Passenger/pillion; ICU: Intensive care unit. ^*^The presented values are adjusted clinical management and outcome frequencies estimated using the “margins” command in STATA.

Table 4 presents the frequency of extremities and pelvis fractures in car and motorcycle crashes, adjusted for age, gender, ISS, safety device, and vehicle type. Notably, pedestrians (0.6%) exhibited a significantly higher frequency of ilium fractures compared to drivers/riders (0.2%) (*P*= 0.017). Pubis fractures were more frequent in pedestrians (3.4%) than in drivers/riders (1.9%) (*P*< 0.010). When adjusting for age, gender, ISS, safety device, and type of vehicle, 3.3% of pedestrians experienced fractures in the proximal humerus, and 1.8% had fractures in the shaft of the ulna. Both frequencies were higher than those observed in drivers/riders (2.3% and 1.1%; *P*= 0.037 and *P*= 0.029, respectively). Furthermore, the frequency of peritrochanteric fractures in pedestrians was 3.7%, significantly surpassing that of 1.9% among passengers/pillions and 1.7% among drivers/riders (*P*< 0.010). Additionally, fractures in the proximal tibia and shaft of the tibia were found in 10.0% and 15.8% of pedestrians, respectively, which were significantly higher than the corresponding frequencies among drivers/riders and passengers/pillions when adjusting for age, gender, ISS, safety device, and type of vehicle (*P*< 0.001). In pedestrians, the frequency of distal tibia fractures was 5.1%, significantly exceeding 3.6% among drivers/riders and 2.7% among passengers/pillions (*P*< 0.010).

**Table 4:** Specific fractures (extremities and pelvis) in car and motorcycle accidents adjusted for age, gender, iss, safety device, and type of vehicle^*^

**Variables**	**Pedestrian**	**Driver/Rider**	**Passenger/Pillion**	**Pairwise comparison**
S32.3 - Fracture of ilium	0.6 (0.3, 1.0)	0.2 (0.1, 0.4)	0.6 (0.2, 0.9)	A > B, C > B
S32.4 - Fracture of acetabulum	2.4 (1.7, 3.1)	3.2 (2.7, 3.7)	4.1 (3.3, 5.0)	C > A
S32.5 - Fracture of pubis	3.4 (2.6, 4.1)	1.9 (1.5, 2.3)	2.5 (1.9, 3.1)	A > B
S42.2 - Fracture of proximal humerus	3.3 (2.5, 4.0)	2.3 (1.9, 2.8)	4.3 (3.5, 5.2)	A > B, C > B
S42.3 - Fracture of shaft of humerus	1.4 (0.9, 1.9)	2.6 (2.1, 3.1)	4.8 (3.9, 5.7)	C > B > A
S42.4 - Fracture of distal humerus	1.5 (1.0, 2.0)	1.3 (1.0, 1.7)	2.7 (2.1, 3.4)	C > A, C > B
S52 - Fracture of forearm	0.1 (-0.0, 0.3)	0.1 (0.0, 0.2)	0.2 (0.0, 0.3)	-
S52.0 - Fracture of proximal of ulna	1.1 (0.6, 1.5)	1.3 (0.9, 1.6)	1.3 (0.8, 1.8)	-
S52.1 - Fracture of proximal radius	0.5 (0.2, 0.8)	0.7 (0.5, 0.9)	0.7 (0.3, 1.1)	-
S52.2 - Fracture of shaft of ulna	1.8 (1.2, 2.4)	1.1 (0.8, 1.4)	2.0 (1.4, 2.7)	A > B, C > B
S52.3 - Fracture of shaft of radius	0.9 (0.5, 1.3)	1.5 (1.2, 1.9)	1.7 (1.2, 2.3)	C > A
S52.4 - Fracture of shafts of both ulna and radius	0.4 (0.1, 0.6)	0.5 (0.3, 0.6)	0.6 (0.3, 1.0)	-
S52.5 - Fracture of distal radius	7.2 (6.1, 8.3)	7.3 (6.6, 8.1)	6.6 (5.5, 7.7)	-
S52.6 - Fracture of distal end of both ulna and radius	0.9 (0.5, 1.3)	1.6 (1.2, 2.0)	1.3 (0.8, 1.7)	B > A
S52.7 - Multiple fractures of the forearm	2.1 (1.5, 2.8)	1.2 (0.9, 1.5)	2.2 (1.5, 2.8)	A > B, C > B
S72 - Fracture of femur	0.1 (-0.0, 0.3)	0.5 (0.3, 0.7)	0.5 (0.2, 0.8)	-
S72.0 - Fracture of neck of femur	2.7 (2.0, 3.4)	2.0 (1.6, 2.4)	2.1 (1.5, 2.7)	-
S72.1 - Peritrochanteric fracture	3.7 (2.9, 4.5)	1.7 (1.3, 2.0)	1.9 (1.3, 2.5)	A > B, A > C
S72.2 - Subtrochanteric fracture	1.3 (0.7, 1.8)	0.9 (0.7, 1.2)	1.1 (0.7, 1.6)	-
S72.3 - Fracture of shaft of femur	4.6 (3.7, 5.6)	6.0 (5.4, 6.6)	6.2 (5.2, 7.1)	B > A, C > A
S72.4 - Fracture of distal femur	2.4 (1.7, 3.0)	2.9 (2.4, 3.4)	2.2 (1.6, 2.9)	-
S72.7 - Multiple fractures of the femur	0.1 (-0.0, 0.2)	0.3 (0.2, 0.4)	0.4 (0.1, 0.7)	-
S82 - Fracture of the lower leg, including ankle	0.4 (0.2, 0.7)	0.4 (0.2, 0.5)	0.2 (0.0, 0.4)	-
S82.0 - Fracture of patella	2.0 (1.3, 2.6)	3.1 (2.6, 3.5)	2.3 (1.6, 2.9)	B > A
S82.1 - Fracture of proximal tibia	10.0 (8.7, 11.3)	7.1 (6.4, 7.8)	4.1 (3.2, 5.0)	A > B > C
S82.2 - Fracture of shaft of tibia	15.8 (14.3, 17.4)	11.8 (10.9, 12.6)	8.6 (7.3, 9.8)	A > B > C
S82.3 - Fracture of distal tibia	5.1 (4.1, 6.0)	3.6 (3.0, 4.1)	2.7 (2.0, 3.3)	A > B, A > C
S82.4 - Fracture of fibula alone	2.8 (2.1, 3.5)	2.2 (1.8, 2.6)	1.1 (0.6, 1.5)	A > C, B > C
S82.5 - Fracture of medial malleolus	3.2 (2.4, 3.9)	2.7 (2.3, 3.1)	1.8 (1.2, 2.4)	A > C, B > C
S82.6 - Fracture of lateral malleolus	2.2 (1.5, 2.8)	2.0 (1.6, 2.4)	1.4 (0.9, 1.9)	-
S82.7 - Multiple fractures of the lower leg	1.3 (0.7, 2.0)	0.3 (0.2, 0.4)	0.8 (0.4, 1.2)	A > B, C > B

*Note*. A: Pedestrian; B: Driver/rider; C: Passenger/pillion; ISS: Injury severity score. ^*^The values presented are adjusted fracture frequencies estimated using the “margins” command in STATA.

 Approximately 1.6% of drivers/riders suffered from distal radius and ulna fractures, surpassing the 0.9% detected in pedestrians (*P*= 0.031). Among drivers/riders, 6.0% represented femur shaft fractures, while 4.6% of pedestrians had this type of fracture (*P*= 0.028). In addition, 3.1% of drivers/riders experienced patella fractures, a statistically significant increase compared to the 2.0% observed in pedestrians (*P*= 0.016).

Table 5 outlines the frequency of neck and spine fractures in car crashes as well as head and face fractures in motorcycle crashes adjusted for age, gender, safety device, and type of accident. Fractures in the cervical vertebrae C3-C7, thoracic vertebrae, and lumbar vertebrae were more frequent in drivers (3.2%, 7.0%, and 8.3%) in comparison to car passengers (1.4%, 4.9%, and 5.6%; *P* = 0.006, *P*= 0.042, and *P*= 0.012, respectively). There were no significant differences in head and face fractures between riders and motorcycle pillions.

**Table 5:** Neck and spine fractures in car accidents and head and face fractures in motorcycle accidents adjusted for age, gender, safety device, and type of accident^*^

**Variables**	**Car crashes**			**Variables**	**Motorcycle crashes**		
	**Driver**	**Passenger**	***P *** **value**		**Rider**	**Pillion**	***P *** **value**
S12.0- Fracture of first cervical vertebra (C1 fracture)	0.6 (0.1, 1.1)	0.5 (0.2, 0.9)	0.758	S02 - Fracture of skull and facial bones	0.6 (0.3, 0.8)	0.5 (-0.2, 1.3)	0.923
S12.1- Fracture of second cervical vertebra (C2 fracture)	0.8 (0.3, 1.2)	1.1 (0.5, 1.7)	0.389	S02.0 - Fracture of vault of skull	1.7 (1.3, 2.1)	2.0 (0.7, 3.2)	0.706
S12.2- Fracture of another specified cervical vertebra	3.2 (2.0, 4.3)	1.4 (0.8, 2.0)	0.006	S02.1 - Fracture of the base of the skull	3.8 (3.1, 4.4)	2.9 (1.4, 4.3)	0.328
S12.7- Multiple fractures of the cervical spine	0.5 (0.1, 0.9)	0.6 (0.2, 1.0)	0.714	S02.2 - Fracture of nasal bones	3.7 (3.1, 4.3)	3.1 (1.6, 4.6)	0.502
S22.0- Fracture of thoracic vertebra	7.0 (5.4, 8.5)	4.9 (3.9, 6.0)	0.042	S02.3 - Fracture of orbital floor	0.8 (0.6, 1.1)	1.0 (0.1, 2.0)	0.661
S22.1- Multiple fractures of the thoracic spine	0.9 (0.4, 1.5)	0.7 (0.2, 1.1)	0.458	S02.4 - Fracture of malar and maxillary bones	4.2 (3.6, 4.8)	2.9 (1.3, 4.4)	0.175
S32.0- Fracture of lumbar vertebra	8.3 (6.6, 10.0)	5.6 (4.5, 6.7)	0.012	S02.6 - Fracture of mandible	2.1 (1.7, 2.5)	1.5 (0.5, 2.6)	0.402
S32.1- Fracture of sacrum	1.6 (0.8, 2.5)	1.5 (0.9, 2.1)	0.845	S02.7 - Multiple fractures involving skull and facial bones	2.1 (1.5, 2.6)	1.0 (0.1, 1.9)	0.160
S32.2- Fracture of coccyx	0.2 (-0.1, 0.5)	0.1 (0.0, 0.2)	0.603				

*Note*. Bold indicates column proportions that differ significantly at the 0.05 level. ^*^The presented values are adjusted fracture frequencies estimated using the “margins” command in STATA.

Tables S1 and S2 (Supplementary file 1) summarize the data related to the distribution of fracture sites in both car and motorcycle crashes.

## Discussion

 This study evaluated 10 114 trauma patients involved in RTCs, with motorcycle crashes being more frequent than car crashes. In car crashes, drivers and passengers had a higher frequency of head, spine, pelvis, and lower extremity fractures, while pedestrians experienced more upper extremity injuries. Similarly, in motorcycle crashes, riders had more head and spine fractures, whereas pedestrians struck by motorcycles had a higher rate of upper extremity fractures. Multiple and comminuted fractures were more common in car crash drivers and motorcycle riders compared to other groups. Spinal surgeries were more frequently required in car crash drivers and motorcycle riders. Fracture patterns varied across groups, with the pelvis, proximal humerus, and tibia fractures being more frequent in pedestrians, while distal radius, femur shaft, and patella fractures were more common in drivers/riders. These findings highlight the disproportionate burden of injuries among motorcycle riders and pedestrians, emphasizing the need for targeted preventive measures and improved trauma care strategies.

 It should be noted that RTCs involving bicycles, pick-up trucks, vans, buses, and heavy transport vehicles were excluded from the analysis because car and motorcycle crashes were the most common types of RTCs in our dataset. Additionally, our study aimed to analyze the pattern of RTC-associated fractures, specifically in drivers, riders, passengers, pillions, and pedestrians. Including other vehicle types might introduce variability in fracture patterns due to differences in impact dynamics, vehicle structures, and protective measures. By focusing solely on car and motorcycle crashes, we ensured a more precise and meaningful analysis of fracture distribution among these distinct groups.

 In both car and motorcycle crashes, males were more commonly involved compared to females. Different patterns of injuries were observed among drivers, passengers, pedestrians, riders, and pillions, with varying frequencies of head injuries, upper and lower extremity injuries, vertebral fractures, and other types of fractures depending on the role in the crash.

 The majority of the RTC-related injured individuals were motorcyclists. Motorcycles have increasingly become the primary mode of transportation in many developing nations, highlighting their growing popularity for intra-city goods and passenger transport or commuting. Despite the surge in motorcycle usage, there is insufficient enforcement of proper rider qualification and licensing procedures. This has led to widespread ignorance and low adherence to traffic laws, risky behaviors among motorcyclists, neglect of road safety practices, and overall increased risk of RTCs.^24^ In a study involving 6306 patients with multiple traumas referred to Hasheminejad University Hospital in Mashhad, Iran, it was found that the most common mechanism of injury was motorcycle crashes.^25^ Brazegar et al demonstrated that a significant portion of fatal traffic injury cases in Iran involved motorcycle users.^26^

 The mean age of those involved in motorcycle accidents (33.7 ± 16.8) was significantly lower than that of those in car accidents (37.3 ± 19.3), which aligns with the findings of other studies. The results of a study by Sadeghi-Bazargani et al on the epidemiological patterns of RTCs in Iran (1996–2014) showed that the majority of motorcycle accident victims were young and adolescent riders, particularly when compared to other age groups.^27^ Based on the findings of Heydari et al, the mean age of motorcycle accident victims in Fars Province (Iran) was 31.4 ± 16.5 years. It was revealed that motorcycle accidents frequently involved younger age groups, specifically those aged 15–35 years.^28^ A cross-sectional study in Sistan and Baluchestan province (Iran) confirmed that more than half of motor riders were aged between 15 years and 30 years.^29^

 Approximately 81.6% and 18.7% of motorcyclists were male and under 18, respectively. This demographic distribution emphasizes the vulnerability of young male motorcyclists in RTC-related trauma. Dos Santos Batista et al conducted a parallel study on 3528 motorcycle crash victims over seven years, with a significant male predominance constituting 88.86% of all victims.^30^ Furthermore, Brockamp et al’s investigation of 24 373 road accident trauma victims from the German trauma registry underscored a heightened frequency of severe and fatal injuries among the younger population.^31^

 Fractures were predominantly observed in the lower extremities, with the tibia emerging as the most frequently fractured bone, followed by the femur in our study. The upper extremities, particularly the radius and humerus, ranked second in common fractures, which is in line with the findings of some other studies.^30,32,33^ A retrospective hospital-based study performed in Kashan (Iran) reported these trends, emphasizing a more significant occurrence of fractures in the lower extremities, with the tibia and fibula taking precedence, followed by the femur.^12^

 In our study, a significant pattern emerged, as head fractures exhibited a higher frequency among drivers than car passengers and pedestrians struck by cars. While the pervasiveness of traffic accidents continues to be a leading cause of facial fractures, our study emphasized an intriguing distinction between drivers and pedestrians struck by cars as well as between riders and motorcycle pillions and pedestrians involved in motorcycle crashes. Drivers might face increased vulnerability due to their proximity to the steering wheel. Fonseca et al found that drivers not wearing seat belts experienced 6.33 fractures per patient, whereas those wearing seat belts had 5.54 fractures per patient.^34^ Modern safety features, mainly steering wheel protection measures, such as airbags, may contribute to a reduction in facial fractures. This implies that while drivers are exposed to potential injuries due to their position, advanced safety technologies in vehicles, especially those protecting the steering wheel, might effectively mitigate them.^34^ The prevalence of such safety features is emphasized, especially in Europe and North America.^35,36^ Our study revealed no cases involving airbag-equipped vehicles, possibly due to their low prevalence in our region or the effective prevention of facial fractures when airbags were activated.

 To further understand the discrepancies noted in injuries between drivers, car passengers, and pedestrians struck by cars, it is essential to consider additional factors, such as airbag deployment and the presence or absence of side impact protection systems. Modern vehicles equipped with steering wheel protection measures, including airbags, may significantly contribute to the protection of drivers. Conversely, the absence of a steering wheel in passenger positions may expose them to increased forward momentum, potentially concentrating force on areas of restraint, particularly the chest and abdomen.^37^ Our study uncovered a contrasting result, showing a higher frequency of thorax injuries in drivers compared to car passengers, which could be attributed to the inadequate use of airbags in our region. Additionally, our results confirmed a higher frequency of spine fractures in drivers compared to car passengers, which could be due to the increased flexion and extension of the spine associated with these types of injuries in drivers. Investigating traffic accident victims, Pigolkin et al found that the driver’s most common fatal injuries consist of fractures in the cervical, thoracic, and lumbar vertebrae, which were much less frequent in passengers seated in the front and right back seats.^38^

 Pedestrians struck by cars demonstrated a higher frequency of proximal tibia, shaft of tibia, and distal tibia fractures in our study, aligning with the notion that the knee joint is one of the most vulnerable body parts in car-to-pedestrian collisions.^39^ The impact, concentrated mainly on the knee joint or just below it, is attributed to the low bumper height in passenger cars.^40^

 Similar findings were noted by Otte et al, who studied lower leg fractures in pedestrians and bicyclists after collisions, revealing a higher prevalence of proximal tibia fractures in pedestrians than in cyclists.^41^ A retrospective study spanning three years further pointed out the significant prevalence of ligamentous knee injuries in pedestrians struck by motor vehicles.^42^

 In the current study, motorcycle riders had a significantly higher incidence of tibia, fibula, and medial malleolus injuries compared to pillions and passengers. Pillion passengers sustained more fractures of the ilium, humerus, and ulna. Zhao et al compared injuries sustained by motorcycle drivers with those sustained by pillion passengers in fatal head-on motorcycle collision accidents and reported similar findings, representing that riders were more likely to suffer lower leg injuries than pillion passengers.^43^

 The findings revealed distinct clinical management and outcome patterns in road users, underscoring the importance of targeted clinical management strategies for different groups involved in RTCs. Pedestrians, who often sustain severe injuries, may benefit from advanced trauma care and rapid surgical interventions to address complex fractures requiring open reduction. Similarly, the higher incidence of spinal repairs among drivers and motorcycle riders necessitates prompt diagnosis and specialized orthopedic and neurosurgical care to optimize outcomes. Chichom-Mefire et al, analyzing 811 cases of road traffic victims and comparing injuries across different road user categories, concluded that pedestrians were significantly more prone to external injuries. In contrast, car occupants were more likely to sustain chest and spine injuries,^44^ which corroborates our findings.

 In our study, when adjusted for age, gender, ISS, safety device, and type of vehicle, ilium and humerus fractures were more prevalent in passengers/pillions compared to drivers/riders, consistent with the findings of other studies. A retrospective study involving 191 patients injured in motor vehicle collisions underscored the higher occurrence of lower limb and pelvis fractures in males, rear seat passengers, and occupants of smaller vehicles.^45^ Notably, pelvis ring fractures were frequently observed when a component of the door or side of the car intruded into the passenger compartment, compressing the pelvis laterally.^46^

 Based on our results, only 1.8% of drivers and 1.6% of car passengers experienced neck injuries, a significantly lower rate compared to what is typically reported in the literature. This discrepancy raises questions about potential factors contributing to our findings, such as the possibility of underdiagnosed injuries or a failure to recognize certain symptoms, which could have resulted in incomplete data in the NTRI dataset. According to previous studies, there is a generally accepted “safe threshold” for rear-end impacts, suggesting that velocities between 10 km/h and 15 km/h should not cause harm.^47^ Individuals involved in rear-end collisions, particularly drivers, face a significantly higher risk of suffering whiplash injuries, and factors such as age and gender seem to play a less significant role.^48^ To ensure the accuracy of our results, we adjusted for variables like age, gender, and safety device usage. Unfortunately, we lacked specific information about factors such as vehicle speed or the nature of the collision (e.g., car-to-car or car-to-wall).

 Our study’s strength is exemplified by its large sample size, allowing for a thorough exploration of distinct fracture types in the Iranian population and providing distinctive insights not present in other studies. The combination of a large sample size, a unique focus on different fracture types, and detailed categorization strengthens our study’s overall impact and relevance, marking it as a valuable addition to the field.

 Acknowledging the limitations of our study is crucial. The data were collected from 12 major trauma centers and thus may not fully represent all trauma centers across the country, potentially limiting the generalizability of our findings. Our study did not have access to detailed data on passenger seat positions, including front, center, and outboard seats. This lack of specific seat position information may influence the assessment of injury patterns, as different seat positions are associated with varying levels of safety and injury outcomes. Additionally, we were unable to obtain data on various vehicle characteristics, such as vehicle type, weight, and speed at the time of the accident. These factors are critical as they significantly impact the severity and pattern of injuries. Our national trauma registry lacks information on trauma victims who succumbed at the scene of the accident. This omission is important as it excludes a subset of the most severe cases from our analysis. We had no access to factors that influence the severity of injuries primarily related to the pre-hospital phase, such as pre-hospital times, patient stabilization and immobilization, underlying comorbidities, vehicle or motorcycle speed at the time of the incident, and the like.

**Highlights**

- In motorcycle crashes, 18.4% of riders were under 18 years old.
- Car drivers experienced the highest rate of head, neck, and thorax injuries.
- Motorcycle riders sustained the most head, face, thorax, and spine injuries.
- Drivers had significantly more C3-C7, thoracic, and lumbar vertebra fractures than car passengers.
- There were no significant differences in head and face fractures between riders and motorcycle pillions.

## Conclusion

 After adjusting for age, gender, and safety device use, distinct injury patterns emerged among different road users in motor vehicle crashes. Drivers had a higher frequency of head, spine, and multiple fractures, often requiring spinal repairs, while pedestrians struck by cars experienced more upper extremity fractures, frequently treated with casts, splints, and open reductions. In motorcycle crashes, riders sustained more head, face, and multiple fractures, leading to higher spinal repair rates, whereas pedestrians struck by motorcycles required more fracture immobilization and closed reductions. Recognizing these injury patterns is crucial for improving prevention strategies, designing protective equipment, and optimizing trauma care protocols to enhance road safety.

## Acknowledgments

 We sincerely appreciate all our colleagues for their great participation in this study.

## Competing Interests

 The authors declare no potential competing interests.

## Ethical Approval

 This study was approved by the Ethics Committee of Sina Hospital, Tehran University of Medical Sciences (Approval ID IR.TUMS.SINAHOSPITAL.REC.1399.090). All methods were performed following the ethical standards as laid down in the Declaration of Helsinki and its later amendments or comparable ethical standards. Written informed consent was obtained from all participants or a parent and/or legal guardian if participants were under 16.

## Funding

 Sina Trauma and Surgery Research Center, Tehran University of Medical Sciences, Tehran, Iran, financially supported the study.

## Supplementary Files

Supplementary file 1 contains Figures S1-S4 and Tables S1-S2.
