# Supplementary material for: The Pattern of Fractures in Road Traffic Crashes: Findings From the National Trauma Registry in Iran
Source: J Res Health Sci. 2025 Jun 10;25(3):e00657. doi: 10.34172/jrhs.8915 (PMC12445884; doi:10.34172/jrhs.8915)
Supplement: Supplementary file 1 — contains Figures S1-S4 and Tables S1-S2. [file jrhs-25-e00657-s001.pdf]

JRHS
Journal of Research in Health Sciences
doi:10.34172/jrhs.8915
JRHS 2025; 25(3):e00657

Supplementary file 1

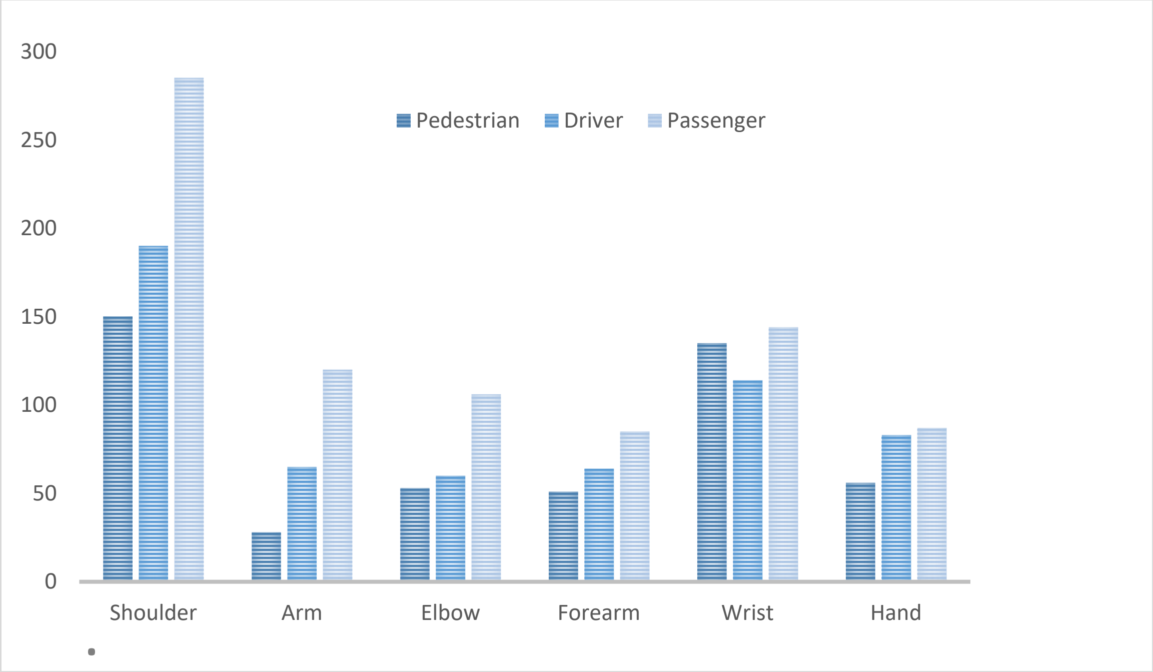

Figure S1. Fractures in only the upper limbs categorized by topographic region in car accidents

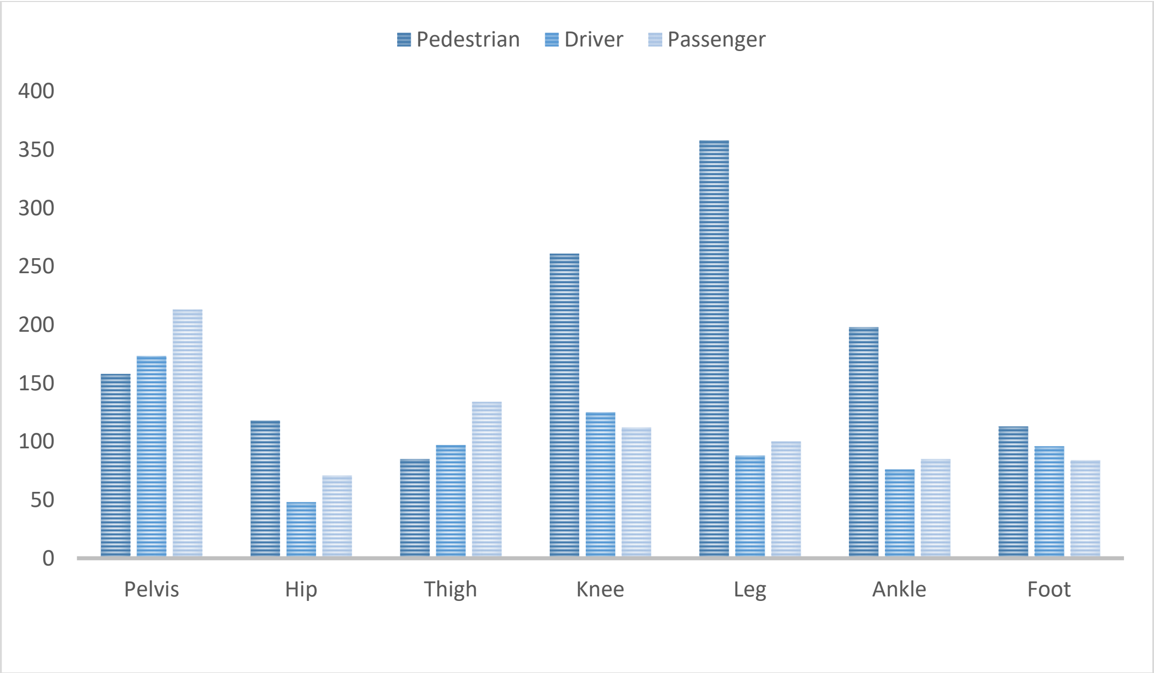

Figure S2. Fractures in only the lower limbs categorized by topographic region in car accidents

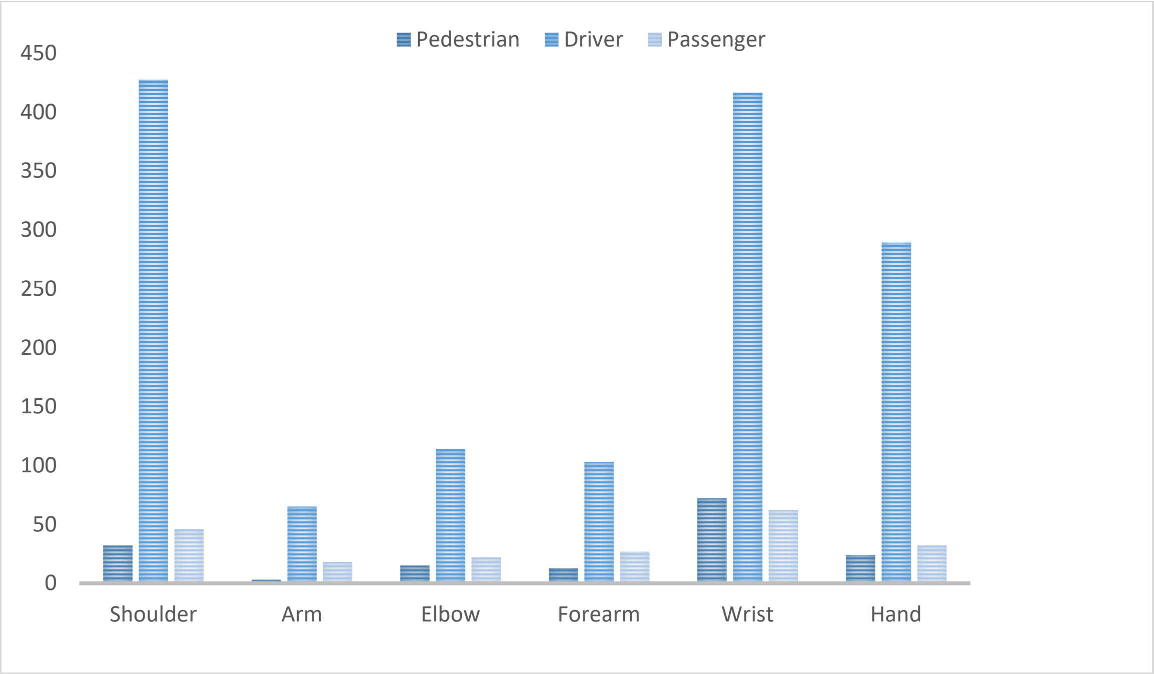

Figure S3. Fractures in only the upper limbs categorized by topographic region in motorcycle accidents

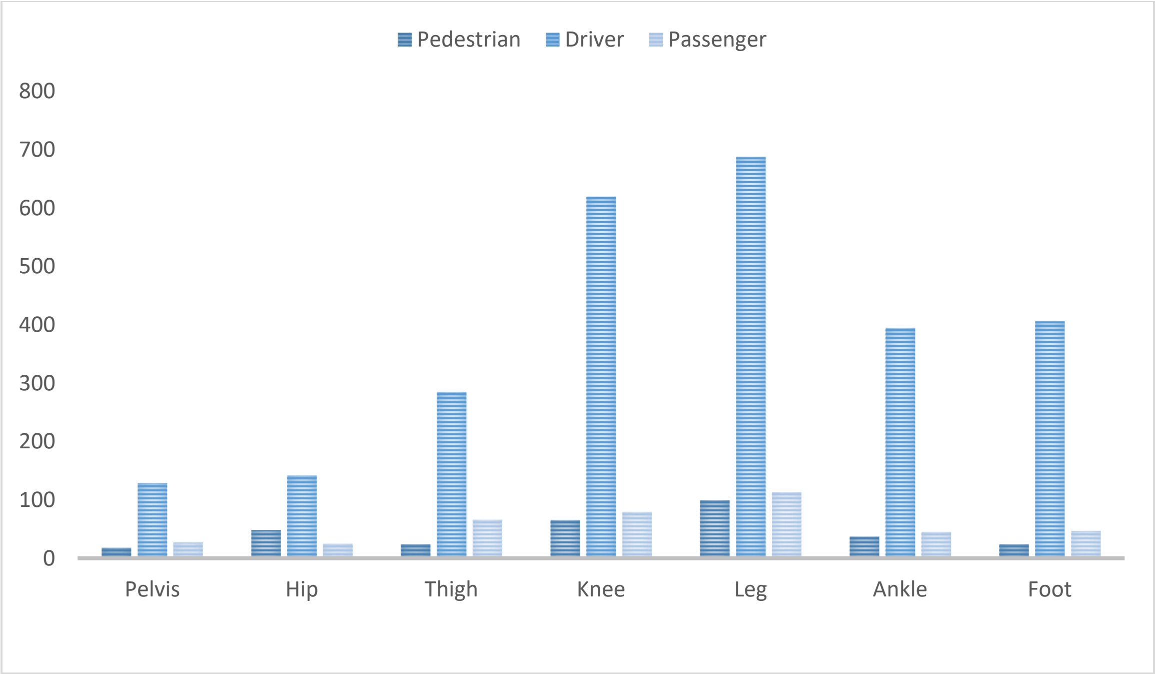

Figure S4. Fractures in only the lower limbs categorized by topographic region in motorcycle accidents.

Table S1. Specific fractures in car crashes.						
	**Pedestrian**	**Driver**	**Passenger**	**Total**	**p-value**	**Pairwise comparison**
**S02 Fracture of skull and facial bones**						
S02 - Fracture of skull and facial bones	2 (0.1)	3 (0.2)	10 (0.6)	15 (0.3)	0.037	-
S02.0 - Fracture of vault of skull	19 (1.1)	27 (2.0)	23 (1.4)	69 (1.5)	0.028	-
S02.1 - Fracture of the base of the skull	29 (1.7)	38 (2.8)	34 (2.0)	101 (2.1)	0.079	-
S02.2 - Fracture of nasal bones	39 (2.3)	68 (5.1)	80 (4.7)	187 (3.9)	<0.001	B>A, C>A
S02.3 - Fracture of orbital floor	6 (0.3)	13 (1.0)	13 (0.8)	32 (0.7)	0.093	-
S02.4 - Fracture of malar and maxillary bones	24 (1.4)	49 (3.7)	50 (3.0)	123 (2.6)	<0.001	B>A, C>A
S02.5 - Fracture of tooth	5 (0.3)	12 (0.9)	18 (1.1)	35 (0.7)	0.022	C>A
S02.6 - Fracture of mandible	18 (1.0)	38 (2.8)	42 (2.5)	98 (2.1)	<0.001	B>A, C>A
S02.7 - Multiple fractures involving skull and facial bones	10 (0.6)	16 (1.2)	14 (0.8)	40 (0.8)	0.178	-
S02.8 - Fractures of other skull and facial bones	14 (0.8)	17 (1.3)	21 (1.2)	52 (1.1)	0.368	-
S02.9 - Fracture of skull and facial bones, part unspecified	24 (1.4)	19 (1.4)	27 (1.6)	70 (1.5)	0.872	-
**S12 Fracture of neck**						
S12 - Fracture of neck	2 (0.1)	0 (0.0)	3 (0.2)	5 (0.1)	0.323	-
S12.0 - Fracture of first cervical vertebra (C1 fracture)	5 (0.3)	7 (0.5)	10 (0.6)	22 (0.5)	0.401	-
S12.1 - Fracture of second cervical vertebra (C2 fracture)	0 (0.0)	13 (1.0)	16 (0.9)	29 (0.6)	<0.001	B>A, C>A
S12.2 - Fracture of other specified cervical vertebra	6 (0.3)	38 (2.8)	26 (1.5)	70 (1.5)	<0.001	B>C>A
S12.7 - Multiple fractures of the cervical spine	0 (0.0)	6 (0.4)	10 (0.6)	16 (0.3)	0.008	B>A, C>A
S12.8 - Fracture of other parts of the neck	1 (0.1)	0 (0.0)	0 (0.0)	1 (0.0)	0.415	-
S12.9 - Fracture of neck, part unspecified	2 (0.1)	13 (1.0)	14 (0.8)	29 (0.6)	0.004	B>A, C>A
**S22 Fracture of rib(s), sternum and thoracic spine**						
S22 – Fracture of rib(s), sternum, and thoracic spine	1 (0.1)	4 (0.3)	5 (0.3)	10 (0.2)	0.224	-

Table S1. Specific fractures in car crashes.						
	**Pedestrian**	**Driver**	**Passenger**	**Total**	**p-value**	**Pairwise comparison**
S22.0 - Fracture of thoracic vertebra	27 (1.6)	84 (6.3)	90 (5.3)	201 (4.2)	<0.001	B>A, C>A
S22.1 - Multiple fractures of the thoracic spine	4 (0.2)	14 (1.0)	10 (0.6)	28 (0.6)	0.014	B>A
S22.2 - Fracture of sternum	2 (0.1)	18 (1.3)	16 (0.9)	36 (0.8)	<0.001	B>A, C>A
S22.3 - Fracture of rib	20 (1.2)	69 (5.2)	55 (3.2)	144 (3.0)	<0.001	B>C>A
S22.4 - Multiple fractures of ribs	14 (0.8)	42 (3.1)	35 (2.1)	91 (1.9)	<0.001	B>A, C>A
S22.8 - Fracture of other parts of bony thorax	0 (0.0)	3 (0.2)	2 (0.1)	5 (0.1)	0.161	-
S22.9 - Fracture of bony thorax, part unspecified	0 (0.0)	0 (0.0)	4 (0.2)	4 (0.1)	0.027	-
**S32 Fracture of lumbar spine and pelvis**						
S32 - Fracture of lumbar spine and pelvis	4 (0.2)	5 (0.4)	1 (0.1)	10 (0.2)	0.166	-
S32.0 - Fracture of lumbar vertebra	38 (2.2)	98 (7.3)	103 (6.1)	239 (5.0)	<0.001	B>A, C>A
S32.1 - Fracture of sacrum	22 (1.3)	17 (1.3)	30 (1.8)	69 (1.5)	0.390	-
S32.2 - Fracture of coccyx	3 (0.2)	2 (0.1)	2 (0.1)	7 (0.1)	0.913	-
S32.3 - Fracture of ilium	12 (0.7)	4 (0.3)	13 (0.8)	29 (0.6)	0.220	-
S32.4 - Fracture of acetabulum	47 (2.7)	101 (7.6)	98 (5.8)	246 (5.2)	<0.001	B>A, C>A
S32.5 - Fracture of pubis	74 (4.3)	49 (3.7)	70 (4.1)	193 (4.1)	0.675	-
S32.7 - Multiple fractures of lumbar spine and pelvis	3 (0.2)	7 (0.5)	7 (0.4)	17 (0.4)	0.244	-
S32.8 - Fracture of other and unspecified parts of lumbar spine and pelvis	9 (0.5)	9 (0.7)	14 (0.8)	32 (0.7)	0.552	-
**S42 Fracture of shoulder and upper arm**						
S42 - Fracture of shoulder and upper arm	1 (0.1)	1 (0.1)	4 (0.2)	6 (0.1)	0.281	-
S42.0 - Fracture of clavicle	66 (3.8)	95 (7.1)	149 (8.8)	310 (6.5)	<0.001	B>A, C>A
S42.1 - Fracture of scapula	23 (1.3)	47 (3.5)	35 (2.1)	105 (2.2)	<0.001	B>C, B>A
S42.2 - Fracture of proximal humerus	61 (3.5)	46 (3.4)	101 (6.0)	208 (4.4)	<0.001	C>A, C>B
S42.3 - Fracture of shaft of humerus	28 (1.6)	65 (4.9)	120 (7.1)	213 (4.5)	<0.001	C>B>A
S42.4 - Fracture of distal humerus	30 (1.7)	26 (1.9)	76 (4.5)	132 (2.8)	<0.001	C>A, C>B

Table S1. Specific fractures in car crashes.						
	**Pedestrian**	**Driver**	**Passenger**	**Total**	**p-value**	**Pairwise comparison**
S42.7 - Multiple fractures of clavicle, scapula, and humerus	0 (0.0)	0 (0.0)	3 (0.2)	3 (0.1)	0.066	-
S42.8 - Fracture of other parts of the shoulder and upper arm	3 (0.2)	3 (0.2)	7 (0.4)	13 (0.3)	0.376	-
S42.9 - Fracture of the shoulder girdle, part unspecified	1 (0.1)	5 (0.4)	6 (0.4)	12 (0.3)	0.130	-
**S52 Fracture of the forearm**						
S52 - Fracture of forearm	2 (0.1)	4 (0.3)	3 (0.2)	9 (0.2)	0.506	-
S52.0 - Fracture of proximal ulna	16 (0.9)	20 (1.5)	22 (1.3)	58 (1.2)	0.339	-
S52.1 - Fracture of proximal radius	7 (0.4)	14 (1.0)	8 (0.5)	29 (0.6)	0.051	-
S52.2 - Fracture of shaft of ulna	29 (1.7)	25 (1.9)	39 (2.3)	93 (2.0)	0.409	-
S52.3 - Fracture of shaft of radius	14 (0.8)	30 (2.2)	34 (2.0)	78 (1.6)	0.003	B>A, C>A
S52.4 - Fracture of shafts of both ulna and radius	8 (0.5)	9 (0.7)	12 (0.7)	29 (0.6)	0.616	-
S52.5 - Fracture of distal radius	111 (6.4)	85 (6.4)	116 (6.8)	312 (6.6)	0.836	-
S52.6 - Fracture of the distal of both ulna and radius	14 (0.8)	18 (1.3)	22 (1.3)	54 (1.1)	0.280	-
S52.7 - Multiple fractures of the forearm	33 (1.9)	15 (1.1)	33 (1.9)	81 (1.7)	0.153	-
S52.8 - Fracture of other parts of the forearm	7 (0.4)	15 (1.1)	13 (0.8)	35 (0.7)	0.070	-
S52.9 - Fracture of the forearm, part unspecified	0 (0.0)	3 (0.2)	7 (0.4)	10 (0.2)	0.031	C>A
**S62 Fracture at wrist and hand level**						
S62 - Fracture at wrist and hand level	4 (0.2)	3 (0.2)	8 (0.5)	15 (0.3)	0.357	-
S62.0 - Fracture of navicular [scaphoid] bone of the hand	10 (0.6)	11 (0.8)	6 (0.4)	27 (0.6)	0.232	-
S62.1 - Fracture of other carpal bone(s)	4 (0.2)	0 (0.0)	1 (0.1)	5 (0.1)	0.112	-
S62.2 - Fracture of the first metacarpal bone	7 (0.4)	12 (0.9)	12 (0.7)	31 (0.7)	0.229	-
S62.3 - Fracture of other metacarpal bone	25 (1.4)	33 (2.5)	41 (2.4)	99 (2.1)	0.070	-
S62.4 - Multiple fractures of metacarpal bones	5 (0.3)	8 (0.6)	6 (0.4)	19 (0.4)	0.379	-
S62.5 - Fracture of thumb	4 (0.2)	2 (0.1)	3 (0.2)	9 (0.2)	0.865	-

Table S1. Specific fractures in car crashes.						
	**Pedestrian**	**Driver**	**Passenger**	**Total**	**p-value**	**Pairwise comparison**
S62.6 - Fracture of other fingers	9 (0.5)	26 (1.9)	23 (1.4)	58 (1.2)	0.001	B>A, C>A
S62.7 - Multiple fractures of fingers	2 (0.1)	2 (0.1)	1 (0.1)	5 (0.1)	0.735	-
S62.8 - Fracture of other and unspecified parts of wrist and hand	3 (0.2)	9 (0.7)	5 (0.3)	17 (0.4)	0.062	-
**S72 Fracture of femur**						
S72 - Fracture of femur	3 (0.2)	6 (0.4)	14 (0.8)	23 (0.5)	0.022	C>A
S72.0 - Fracture of neck of femur	47 (2.7)	25 (1.9)	39 (2.3)	111 (2.3)	0.299	-
S72.1 – Peritrochanteric fracture	71 (4.1)	23 (1.7)	32 (1.9)	126 (2.6)	<0.001	A>B, A>C
S72.2 - Subtrochanteric fracture	15 (0.9)	21 (1.6)	25 (1.5)	61 (1.3)	0.157	-
S72.3 - Fracture of shaft of femur	70 (4.1)	76 (5.7)	109 (6.4)	255 (5.4)	0.007	C>A
S72.4 - Fracture of distal femur	43 (2.5)	29 (2.2)	42 (2.5)	114 (2.4)	0.815	-
S72.7 - Multiple fractures of the femur	2 (0.1)	6 (0.4)	6 (0.4)	14 (0.3)	0.205	-
S72.8 - Fractures of other parts of the femur	2 (0.1)	1 (0.1)	0 (0.0)	3 (0.1)	0.394	-
S72.9 - Fracture of the femur, part unspecified	12 (0.7)	6 (0.4)	13 (0.8)	31 (0.7)	0.536	-
**S82 Fracture of the lower leg, including ankle**						
S82 - Fracture of the lower leg, including ankle	11 (0.6)	5 (0.4)	3 (0.2)	19 (0.4)	0.101	-
S82.0 - Fracture of patella	29 (1.7)	51 (3.8)	26 (1.5)	106 (2.2)	<0.001	B>A, B>C
S82.1 - Fracture of proximal tibia	189 (89.0)	45 (3.4)	44 (2.6)	278 (5.8)	<0.001	A>B, A>C
S82.2 - Fracture of shaft of tibia	300 (17.4)	64 (4.8)	86 (5.1)	450 (9.5)	<0.001	A>B, A>C
S82.3 - Fracture of distal tibia	93 (5.4)	27 (2.0)	43 (2.5)	163 (3.4)	<0.001	A>B, A>C
S82.4 - Fracture of fibula alone	58 (3.4)	24 (1.8)	14 (0.8)	96 (2.0)	<0.001	A>B, A>C
S82.5 - Fracture of medial malleolus	63 (3.7)	30 (2.2)	25 (1.5)	118 (2.5)	<0.001	A>C
S82.6 - Fracture of lateral malleolus	42 (2.4)	19 (1.4)	17 (1.0)	78 (1.6)	0.003	A>C
S82.7 - Multiple fractures of the lower leg	17 (1.0)	3 (0.2)	14 (0.8)	34 (0.7)	0.037	A>C
S82.8 - Fractures of other parts of the lower leg	82 (4.8)	17 (1.3)	22 (1.3)	121 (2.5)	<0.001	A>B, A>C

Table S1. Specific fractures in car crashes.

	Pedestrian	Driver	Passenger	Total	p-value	Pairwise comparison
S82.9 - Fracture of the lower leg, part unspecified	49 (2.8)	5 (0.4)	5 (0.3)	59 (1.2)	<0.001	A>B, A>C
**S92 Fracture of the foot, except ankle**						
S92 - Fracture of the foot, except ankle	2 (0.1)	1 (0.1)	3 (0.2)	6 (0.1)	0.725	-
S92.0 - Fracture of calcaneus	19 (1.1)	23 (1.7)	11 (0.6)	53 (1.1)	0.020	B>C
S92.1 - Fracture of talus	14 (0.8)	27 (2.0)	23 (1.4)	64 (1.3)	0.016	B>A
S92.2 - Fracture of other tarsal bone(s)	7 (0.4)	12 (0.9)	7 (0.4)	26 (0.5)	0.121	-
S92.3 - Fracture of metatarsal bone	47 (2.7)	25 (1.9)	29 (1.7)	101 (2.1)	0.091	-
S92.4 - Fracture of the great toe	7 (0.4)	2 (0.1)	5 (0.3)	14 (0.3)	0.431	-
S92.5 - Fracture of other toes	16 (0.9)	5 (0.4)	5 (0.3)	26 (0.5)	0.026	-
S92.7 - Multiple fractures of the foot	1 (0.1)	1 (0.1)	1 (0.1)	3 (0.1)	0.980	-
S92.9 - Fracture of the foot, unspecified	5 (0.3)	1 (0.1)	0 (0.0)	6 (0.1)	0.048	-

Table S2. Specific fractures in motorcycle crashes.						
	**Pedestrian**	**Rider**	**Pillion**	**Total**	**p-value**	**Pairwise comparison**
**S02 Fracture of skull and facial bones**						
S02 - Fracture of skull and facial bones	2 (0.4)	22 (0.5)	3 (0.5)	27 (0.5)	0.913	-
S02.0 - Fracture of vault of skull	3 (0.6)	72 (1.7)	13 (2.0)	88 (1.6)	0.113	-
S02.1 - Fracture of the base of the skull	9 (1.7)	154 (3.7)	21 (3.3)	184 (3.4)	0.077	-
S02.2 - Fracture of nasal bones	10 (1.9)	152 (3.6)	21 (3.3)	183 (3.4)	0.140	-
S02.3 - Fracture of orbital floor	2 (0.4)	34 (0.8)	5 (0.8)	41 (0.8)	0.584	-
S02.4 - Fracture of malar and maxillary bones	9 (1.7)	181 (4.3)	17 (2.7)	207 (3.9)	0.004	B>A
S02.5 - Fracture of tooth	3 (0.6)	22 (0.5)	3 (0.5)	28 (0.5)	0.968	-
S02.6 - Fracture of mandible	5 (1.0)	88 (2.1)	10 (1.6)	103 (1.9)	0.170	-
S02.7 - Multiple fractures involving skull and facial bones	4 (0.8)	74 (1.8)	7 (1.1)	85 (1.6)	0.139	-
S02.8 - Fractures of other skull and facial bones	3 (0.6)	74 (1.8)	3 (0.5)	80 (1.5)	0.009	B>C
S02.9 - Fracture of skull and facial bones, part unspecified	4 (0.8)	38 (0.9)	6 (0.9)	48 (0.9)	0.950	-
**S12 Fracture of neck**						
S12 - Fracture of neck	0 (0.0)	0 (0.0)	0 (0.0)	0 (0.0)	-	-
S12.0 - Fracture of first cervical vertebra (C1 fracture)	0 (0.0)	3 (0.1)	1 (0.2)	4 (0.1)	0.615	-
S12.1 - Fracture of second cervical vertebra (C2 fracture)	0 (0.0)	8 (0.2)	3 (0.5)	11 (0.2)	0.191	-
S12.2 - Fracture of other specified cervical vertebra	1 (0.2)	27 (0.6)	2 (0.3)	30 (0.6)	0.295	-
S12.7 - Multiple fractures of the cervical spine	0 (0.0)	3 (0.1)	0 (0.0)	3 (0.1)	0.663	-
S12.8 - Fracture of other parts of the neck	0 (0.0)	1 (0.0)	0 (0.0)	1 (0.0)	0.872	-
S12.9 - Fracture of neck, part unspecified	0 (0.0)	10 (0.2)	0 (0.0)	10 (0.2)	0.254	-
**S22 Fracture of rib(s), sternum and thoracic spine**						

Table S2. Specific fractures in motorcycle crashes.						
	**Pedestrian**	**Rider**	**Pillion**	**Total**	**p-value**	**Pairwise comparison**
S22 – Fracture of rib(s), sternum, and thoracic spine	0 (0.0)	2 (0.0)	1 (0.2)	3 (0.1)	0.470	-
S22.0 - Fracture of thoracic vertebra	1 (0.2)	82 (1.9)	10 (1.6)	93 (1.7)	0.015	B>A
S22.1 - Multiple fractures of the thoracic spine	0 (0.0)	5 (0.1)	2 (0.3)	7 (0.1)	0.306	-
S22.2 - Fracture of sternum	0 (0.0)	3 (0.1)	0 (0.0)	3 (0.1)	0.663	-
S22.3 - Fracture of rib	1 (0.2)	72 (1.7)	4 (0.6)	77 (1.4)	0.005	B>A
S22.4 - Multiple fractures of ribs	2 (0.4)	50 (1.2)	3 (0.5)	55 (1.0)	0.079	-
S22.8 - Fracture of other parts of bony thorax	0 (0.0)	0 (0.0)	0 (0.0)	0 (0.0)	-	-
S22.9 - Fracture of bony thorax, part unspecified	0 (0.0)	1 (0.0)	0 (0.0)	1 (0.0)	0.872	-
**S32 Fracture of lumbar spine and pelvis**						
S32 - Fracture of lumbar spine and pelvis	0 (0.0)	2 (0.0)	1 (0.2)	3 (0.1)	0.470	-
S32.0 - Fracture of lumbar vertebra	5 (1.0)	110 (2.6)	22 (3.5)	137 (2.6)	0.025	C>A
S32.1 - Fracture of sacrum	4 (0.8)	8 (0.2)	6 (0.9)	18 (0.3)	0.002	A>B, C>B
S32.2 - Fracture of coccyx	0 (0.0)	0 (0.0)	1 (0.2)	1 (0.0)	0.024	C>B
S32.3 - Fracture of ilium	1 (0.2)	10 (0.2)	2 (0.3)	13 (0.2)	0.909	-
S32.4 - Fracture of acetabulum	4 (0.8)	75 (1.8)	8 (1.3)	87 (1.6)	0.172	-
S32.5 - Fracture of pubis	9 (1.7)	36 (0.9)	10 (1.6)	55 (1.0)	0.058	-
S32.7 - Multiple fractures of lumbar spine and pelvis	1 (0.2)	8 (0.2)	0 (0.0)	9 (0.2)	0.545	-
S32.8 - Fracture of other and unspecified parts of lumbar spine and pelvis	2 (0.4)	10 (0.2)	1 (0.2)	13 (0.2)	0.725	-
**S42 Fracture of shoulder and upper arm**						
S42 - Fracture of shoulder and upper arm	2 (0.4)	1 (0.0)	0 (0.0)	3 (0.1)	0.004	A>B
S42.0 - Fracture of clavicle	18 (3.5)	287 (6.8)	26 (4.1)	331 (6.2)	<0.001	B>A, B>C
S42.1 - Fracture of scapula	0 (0.0)	65 (1.5)	5 (0.8)	70 (1.3)	0.007	B>A
S42.2 - Fracture of proximal humerus	14 (2.7)	75 (1.8)	15 (2.4)	104 (1.9)	0.252	-

Table S2. Specific fractures in motorcycle crashes.						
	**Pedestrian**	**Rider**	**Pillion**	**Total**	**p-value**	**Pairwise comparison**
S42.3 - Fracture of shaft of humerus	3 (0.6)	65 (1.5)	18 (2.8)	86 (1.6)	0.008	C>A
S42.4 - Fracture of distal humerus	3 (0.6)	38 (0.9)	8 (1.3)	49 (0.9)	0.481	-
S42.7 - Multiple fractures of clavicle, scapula, and humerus	0 (0.0)	1 (0.0)	0 (0.0)	1 (0.0)	0.872	-
S42.8 - Fracture of other parts of the shoulder and upper arm	0 (0.0)	5 (0.1)	0 (0.0)	5 (0.1)	0.504	-
S42.9 - Fracture of the shoulder girdle, part unspecified	0 (0.0)	5 (0.1)	0 (0.0)	5 (0.1)	0.504	-
**S52 Fracture of the forearm**						
S52 - Fracture of forearm	0 (0.0)	1 (0.0)	1 (0.2)	2 (0.0)	0.240	-
S52.0 - Fracture of proximal ulna	8 (1.6)	49 (1.2)	9 (1.4)	66 (1.2)	0.683	-
S52.1 - Fracture of proximal radius	4 (0.8)	27 (0.6)	5 (0.8)	36 (0.7)	0.876	-
S52.2 - Fracture of shaft of ulna	7 (1.4)	37 (0.9)	9 (1.4)	53 (1.0)	0.300	-
S52.3 - Fracture of shaft of radius	6 (1.2)	50 (1.2)	14 (2.2)	70 (1.3)	0.106	-
S52.4 - Fracture of shafts of both ulna and radius	0 (0.0)	16 (0.4)	4 (0.6)	20 (0.4)	0.217	-
S52.5 - Fracture of distal radius	61 (11.8)	302 (7.2)	46 (7.2)	409 (7.6)	<0.001	A>B, A>C
S52.6 - Fracture of the distal of both ulna and radius	9 (1.7)	65 (1.5)	11 (1.7)	85 (1.6)	0.899	-
S52.7 - Multiple fractures of the forearm	11 (2.1)	55 (1.3)	15 (2.4)	81 (1.5)	0.062	-
S52.8 - Fracture of other parts of the forearm	2 (0.4)	32 (0.8)	9 (1.4)	43 (0.8)	0.122	-
S52.9 - Fracture of the forearm, part unspecified	0 (0.0)	2 (0.0)	2 (0.3)	4 (0.1)	0.058	-
**S62 Fracture at wrist and hand level**						-
S62 - Fracture at wrist and hand level	1 (0.2)	10 (0.2)	1 (0.2)	12 (0.2)	0.912	-
S62.0 - Fracture of navicular [scaphoid] bone of the hand	2 (0.4)	49 (1.2)	5 (0.8)	56 (1.0)	0.207	-
S62.1 - Fracture of other carpal bone(s)	0 (0.0)	6 (0.1)	0 (0.0)	6 (0.1)	0.439	-

Table S2. Specific fractures in motorcycle crashes.						
	**Pedestrian**	**Rider**	**Pillion**	**Total**	**p-value**	**Pairwise comparison**
S62.2 - Fracture of the first metacarpal bone	6 (1.2)	19 (0.5)	1 (0.2)	26 (0.5)	0.040	-
S62.3 - Fracture of other metacarpal bone	16 (3.1)	150 (3.6)	21 (3.3)	187 (3.5)	0.831	-
S62.4 - Multiple fractures of metacarpal bones	1 (0.2)	21 (0.5)	2 (0.3)	24 (0.4)	0.535	-
S62.5 - Fracture of thumb	0 (0.0)	14 (0.3)	1 (0.2)	15 (0.3)	0.331	-
S62.6 - Fracture of other fingers	1 (0.2)	75 (1.8)	6 (0.9)	82 (1.5)	0.009	B>A
S62.7 - Multiple fractures of fingers	0 (0.0)	4 (0.1)	1 (0.2)	5 (0.1)	0.683	-
S62.8 - Fracture of other and unspecified parts of wrist and hand	1 (0.2)	11 (0.3)	0 (0.0)	12 (0.2)	0.424	-
**S72 Fracture of femur**						-
S72 - Fracture of femur	0 (0.0)	18 (0.4)	0 (0.0)	18 (0.3)	0.084	-
S72.0 - Fracture of neck of femur	19 (3.7)	76 (1.8)	16 (2.5)	111 (2.1)	0.013	A>B
S72.1 – Peritrochanteric fracture	29 (5.6)	66 (1.6)	9 (1.4)	104 (1.9)	<0.001	A>B, A>C
S72.2 - Subtrochanteric fracture	8 (1.6)	32 (0.8)	5 (0.8)	45 (0.8)	0.177	-
S72.3 - Fracture of shaft of femur	16 (3.1)	253 (6.0)	61 (9.6)	330 (6.2)	<0.001	C>B>A
S72.4 - Fracture of distal femur	8 (1.6)	123 (2.9)	19 (3.0)	150 (2.8)	0.194	-
S72.7 - Multiple fractures of the femur	0 (0.0)	12 (0.3)	1 (0.2)	13 (0.2)	0.414	-
S72.8 - Fractures of other parts of the femur	0 (0.0)	2 (0.0)	0 (0.0)	2 (0.0)	0.760	-
S72.9 - Fracture of the femur, part unspecified	4 (0.8)	21 (0.5)	5 (0.8)	30 (0.6)	0.524	-
**S82 Fracture of the lower leg, including ankle**						-
S82 - Fracture of the lower leg, including ankle	0 (0.0)	16 (0.4)	1 (0.2)	17 (0.3)	0.261	-
S82.0 - Fracture of patella	11 (2.1)	134 (3.2)	20 (3.1)	165 (3.1)	0.423	-
S82.1 - Fracture of proximal tibia	46 (8.9)	362 (8.6)	40 (6.3)	448 (8.4)	0.129	-

Table S2. Specific fractures in motorcycle crashes.						
	**Pedestrian**	**Rider**	**Pillion**	**Total**	**p-value**	**Pairwise comparison**
S82.2 - Fracture of shaft of tibia	66 (12.8)	595 (14.1)	102 (16.0)	763 (14.2)	0.272	-
S82.3 - Fracture of distal tibia	21 (4.1)	171 (4.1)	18 (2.8)	210 (3.9)	0.321	-
S82.4 - Fracture of fibula alone	13 (2.5)	92 (2.2)	11 (1.7)	116 (2.2)	0.642	-
S82.5 - Fracture of medial malleolus	7 (1.4)	132 (3.1)	11 (1.7)	150 (2.8)	0.015	-
S82.6 - Fracture of lateral malleolus	9 (1.7)	91 (2.2)	16 (2.5)	116 (2.2)	0.670	-
S82.7 - Multiple fractures of the lower leg	3 (0.6)	17 (0.4)	5 (0.8)	25 (0.5)	0.387	-
S82.8 - Fractures of other parts of the lower leg	34 (6.6)	147 (3.5)	21 (3.3)	202 (3.8)	0.002	A>B, A>C
S82.9 - Fracture of the lower leg, part unspecified	23 (4.5)	138 (3.3)	16 (2.5)	177 (3.3)	0.183	-
**S92 Fracture of the foot, except ankle**						-
S92 - Fracture of the foot, except ankle	0 (0.0)	7 (0.2)	2 (0.3)	9 (0.2)	0.431	-
S92.0 - Fracture of calcaneus	6 (1.2)	43 (1.0)	5 (0.8)	54 (1.0)	0.800	-
S92.1 - Fracture of talus	2 (0.4)	57 (1.4)	5 (0.8)	64 (1.2)	0.097	-
S92.2 - Fracture of other tarsal bone(s)	0 (0.0)	25 (0.6)	1 (0.2)	26 (0.5)	0.083	-
S92.3 - Fracture of metatarsal bone	13 (2.5)	201 (4.8)	25 (3.9)	239 (4.5)	0.050	-
S92.4 - Fracture of the great toe	0 (0.0)	17 (0.4)	4 (0.6)	21 (0.4)	0.227	-
S92.5 - Fracture of other toes	1 (0.2)	51 (1.2)	3 (0.5)	55 (1.0)	0.032	-
S92.7 - Multiple fractures of the foot	1 (0.2)	2 (0.0)	1 (0.2)	4 (0.1)	0.372	-
S92.9 - Fracture of the foot, unspecified	1 (0.2)	3 (0.1)	1 (0.2)	5 (0.1)	0.590	-
